# Comparative analysis of the phenolic contents and antioxidant activities of different parts of two pomegranate (*Punica granatum* L.) Cultivars: ‘Tunisia’ and ‘Qingpi’

**DOI:** 10.3389/fpls.2023.1265018

**Published:** 2023-09-29

**Authors:** Huifen Zhang, Miao Wang, Guoqiang Yu, Jing Pu, Kun Tian, Xiaofu Tang, Ying Du, Hongxia Wu, Jiong Hu, Xian Luo, Lijin Lin, Qunxian Deng

**Affiliations:** ^1^ College of Horticulture, Sichuan Agricultural University, ChengDu, Sichuan, China; ^2^ Rural Professional Technology Association of Huili, Huili, Sichuan, China; ^3^ Science and Technology Association of Huili, Huili, Sichuan, China

**Keywords:** *Punica granatum* L., phenolic contents, antioxidant activity, different parts, cultivars

## Abstract

Pomegranate (*Punica granatum* L.), with its abundant phenolic substances and strong antioxidant activity, holds significant research and utilization potential across various organs. However, there have been few studies on the phenolic content and antioxidant activity of different parts of pomegranate, especially the placenta. This study investigated the phenolic content and antioxidant activity of fruits, flowers, and leaves of two pomegranate varieties, ‘Tunisia’ and ‘Qingpi’, throughout their growth and development. Results indicated significant variations in phenolic content among different organs, with petals exhibiting the highest total polyphenol content (TPC, 49.40 mg GAE/g FW) and total anthocyanin content (TMAC, 1938.54 nmol/g FW). Placenta contained the highest levels of total flavonoids (TFC, 173.58 mg RE/g FW) and punicalagin (109.30 mg/g FW). The peel had the highest content of total flavanols (TFAC, 19.42 mg CE/g FW). Over the course of pomegranate development, total polyphenols, total flavonoids, total flavanols, punicalagin, and antioxidant activity declined in different organs. Antioxidant activity followed the order: fruit > flower > leaf, with the placenta exhibiting the highest antioxidant activity among fruits. Antioxidant activity showed a significant positive correlation with total polyphenols (R^2 = ^0.77-1.00), total flavonoids (R^2 = ^0.71-0.99, except tegmens), and punicalagin (R^2 = ^0.71-1.00). This study provides a comparative analysis of the phenolic content and antioxidant activity in different organs of pomegranate, highlighting the placenta as the primary source of punicalagin. This study provides a theoretical basis for the development and utilization of pomegranate phenolic compounds.

## 1 Introduction

Pomegranate (*Punica granatum* L.) is a small arbor or shrub belonging to the family Lythraceae. It is used for both food and medicinal purposes (Zhao and Yuan, 2021; Wang et al., 2023). Pomegranate is known for its strong antioxidant properties due to its abundant phenolic compounds, and it has been traditionally used for preventing and treating various diseases, such as heart disease and diabetes (Asgary et al., 2014; Bahmani et al., 2014; Asgharpour and Alirezaei, 2021). Natural extracts from pomegranate are also widely used in food products as antioxidants and antimicrobial agents (Shaygannia et al., 2016; Martínez et al., 2019; Essid et al., 2020; Parafati et al., 2021), thus promoting greater research attention on antioxidants and application of pomegranates.

The antioxidant activity of pomegranate is attributed to its phenolic substances, including polyphenols, flavonoids, flavanols, and anthocyanins (Gözlekçi et al., 2011; Ávila-Gálvez et al., 2019; Yuan et al., 2022). Flavonoids scavenge oxygen free radicals, prevent cell degradation, and possess anti-aging properties (Hostetler et al., 2017; Ganai et al., 2021). Flavanols exhibit antioxidant, anti-tumor, anti-inflammatory, and antimicrobial effects (Kopustinskiene et al., 2020; Chagas et al., 2022). Anthocyanins, on the other hand, are edible polyphenolic pigments with strong free radical scavenging ability and anti-cancer properties (Silva et al., 2017; Wang et al., 2023). Indicators such as total polyphenol content (TPC), total flavonoid content (TFC), total flavanol content (TFAC), and total anthocyanin content (TMAC) are often used to assess the antioxidant properties of plants. Pomegranate peel, tegmen, juice, flower, and leaf are rich sources of phenolic substances (Marcelino et al., 2023). Man et al. (2022). identified 64 phenolic compounds in the pomegranate peel. Pomegranate flowers contain polyphenols, flavonoids, and triterpenes (Liu and Seeram, 2018; Niknam et al., 2021; Yisimayili and Chao, 2022), while pomegranate leaves have been found to contain various compounds such as flavonoids, quercetin, tannic acid, ursolic acid, and ellagic tannins (Hussein et al., 1997). Punicalagin is the most abundant polyphenol in pomegranate (Seeram et al., 2005), and it is found in pomegranate fruit as well as in the leaves of *Terminalia chebula* Retz. and *Terminalia catappa* L. (Chen et al., 2009; Mo et al., 2019). Gull et al. (2021) found that the punicalagin content was related to the antioxidant capacity.

Pomegranates are commercially cultivated in over 30 countries, including India, Iran, Spain, China, and the United States (Jaime et al., 2013; Derakhshan et al., 2018). China is one of the largest pomegranate producers, with cultivation dating back over 2000 years (Zhao et al., 2015). Huili is a major pomegranate-producing area in China, with regions such as Huili, Kaifeng, Xingyang, Yicheng, Huaiyuan, Lintong, Mengzi, and Yecheng being the primary distribution areas (Peng et al., 2020). However, there is a lack of studies on the nutritional and functional substances of the main pomegranate varieties in Huili, as well as product development. Although some studies have investigated changes in phenolic content and antioxidant activity during the development of pomegranate fruits, flowers, and leaves (Gözlekçi et al., 2011; Bekir et al., 2013a; Bekir et al., 2013b), there is limited research on the phenolic content and antioxidant activity in different parts of pomegranate. In particular, there is a lack of understanding of phenolic content in placenta. Therefore, this study aims to explore the changes in phenolic content and antioxidant activity during the development of different parts of pomegranate fruits, flowers, and leaves using the ‘Tunisia’ and ‘Qingpi’ pomegranate cultivars, which are prevalent in Huili. The findings of this study can provide valuable insights for the utilization and processing of pomegranate.

## 2 Materials and methods

### 2.1 Plant material

The materials used in this study were 7-year-old ‘Qingpi’ (identified by Pomegranate Research Institute, Huili city, Sichuan Province, China) pomegranate trees and 6-year-old ‘Tunisia’ (identified by Zhongfu Liu, Henan Provincial Economic Forestry and Tree Seedling Workstation, Henan Province, China) trees grafted onto ‘Qingpi’. These trees were located in Zhangguan Town, Huili City, Sichuan Province (26° 51′ N, 102° 29′ E, altitude 1780 m). Leaf samples without obvious pests and diseases in the middle of the branches were collected at three different stages: young leaf expansion (T1, March 21, 2021), turning green (T2, April 5, 2021), and mature (T3, April 21, 2021). For flower samples, both normal and abnormal flowers without diseases or insect pests were collected (April 23, 2021) during the bud period and full-blossom period. The calyx, petals, stamens, and ovaries were sampled separately. The peel, placenta, septum, tegmen, and testa (Juice source) of fruits were sampled 7 times (Samples have been taken every 15 days starting May 28, 2021) from the young fruit stage to the mature fruit stage, including at different stages: 30, 45, 60, 75, 90, 105, and 120 days after flowering (DAF) (S1, S2, S3, S4, S5, S6, and S7, respectively). More than thirty leaves, flowers and fruits of both pomegranate varieties were collected with three biological replicates. Samples were immediately transported to the laboratory, pre-cooled with liquid nitrogen, and stored at -80°C for further analysis.

### 2.2 Extraction and determination of phenolic compounds

#### 2.2.1 Extraction of phenolic compounds

TPC, TFC, and TFAC extraction followed the method by Ozgen et al. (2008). The extraction solution consisted of 70% methanol, 28% anhydrous ethanol, and 2% formic acid (*v/v*). A 0.2 g sample was ground with liquid nitrogen and placed in a centrifuge tube. Subsequently, 5 mL of the extraction solution was added, followed by 30 min of ultrasonic extraction. After shaking at 250 rpm at 30°C for 2 h, the extract was centrifuged at 8000 rpm for 10 min at 4°C. The supernatant was then filtered through a 0.45 μm needle tube filter for analysis of TPC, TFC, TFAC, DPPH, and FRAP. All procedures were conducted in the dark.

#### 2.2.2 Determination of total polyphenol content

The TPC was determined using the Folin-Ciocalteu method (García-Ruiz et al., 2011). For the analysis, 100 μL of the extract was mixed with 1.5 mL of distilled water and 0.1 mL of Folin-Ciocalteu reagent in a centrifuge tube. After shaking well, 1.5 mL of a 20% saturated Na_2_CO_3_ solution was added, and the mixture was shaken again and left to react away from light for 2 h. The absorbance was measured at 765 nm using a spectrophotometer (UV-1800PC, MAPADA Instrument Co., LTD., Shanghai, China). The TPC content was calculated using Gallic Acid Equivalents (GAE) as the standard (50–1000 mg/L).

#### 2.2.3 Determination of total flavonoid content

The TFC was assessed as described by Park et al. (2008). A 200 μL sample extract was mixed with 1.3 mL of methanol, followed by the addition of 100 μL NaNO_2_ (0.5 M) and 100 μL AlCl_3_ (0.3 M). After 5 min of incubation, 500 μL NaOH (1 M) was added. The extraction solvent was used as the control, with the absorbance being measured by a spectrophotometer (UV-1800PC, MAPADA Instrument Co., LTD., Shanghai, China) at 510 nm wavelength, and the content was calculated with rutin (Rutin Equivalents, RE) as the standard (20–100 mg/L).

#### 2.2.4 Determination of total flavanol content

The TFAC was measured following Zhang et al.’s method (Zhang et al., 2023). The extract (100 μL) was sequentially mixed with distilled water (1.5 mL) and 1% p-DMACA solution (1 mL). After 10 min of shaking, the absorbance was measured at 640 nm wavelength using a spectrophotometer (UV-1800PC, MAPADA Instrument Co., LTD., Shanghai, China), with the extraction solvent as the control. The content was determined using catechin as the standard (6.25-200 mg/L) and expressed as catechin equivalents (CE).

#### 2.2.5 Determination of total anthocyanin content

The method by Zhang et al. (2022) was modified to determine TMAC. For this, 0.2 g of the sample or 0.2 mL of fruit juice was added to a centrifuge tube containing 5 mL of a 1% hydrochloric acid-methanol solution. The mixture was shaken and then incubated at 4°C in the dark for 20 h. Ultrasonic extraction was performed for 30 min, followed by centrifugation at 8000 rpm and 4°C for 5 min. The resulting supernatant was collected, and the optical density (OD) was measured at 530 nm, 620 nm, and 650 nm using a microplate reader (Varioskan LUX, Thermo Fisher Scientific, MA, USA). The ODλ value of anthocyanin was calculated as ODλ = (OD_530_ - OD_620_) - 0.1(OD_650_ - OD_620_). Finally, TMAC was calculated using the following formula:


$$\text{TMAC}\left(\text{nmol }/\text{ g}\right)\;=\;\left(\text{ODλ }\times V\times \;10^{6}\right)\;/\;(\text{ϵλ}\times m)$$

V:the total volume of the extract (mL);

ϵλ: molar extinction coefficient of anthocyanin;

m: the amount of sample taken (g or mL)

#### 2.2.6 Determination of punicalagin content

The punicalagin content was quantitatively determined using the high-performance liquid chromatography (HPLC) method described by Zhao et al. (2015). A 0.2 g sample was ground and mixed with 5 mL of extraction solution (methanol: water *v/v* = 84:16) in a centrifuge tube. The tube was shaken and then subjected to ultrasonication for 30 min at room temperature. The mixture was further shaken at 30°C and 250 rpm for 2 h. After centrifugation at 8000 rpm for 15 min at 4°C, the supernatant was filtered through a 0.22 μm microporous filter membrane.

The analysis was performed using an Agilent 1260 series HPLC system (Agilent Technologies, USA) equipped with a Comatex C18 column (5 μm, 46 mm * 250 mm). The column temperature was maintained at 30°C, and 20 μL of the sample was injected. The mobile phase comprised ultrapure water with 0.1% formic acid (A) and acetonitrile chromatographic pure solution with 0.1% formic acid (B). The gradient elution method was used with a flow rate of 1 mL/min. The elution program wa: 0–24 min, 91.8% A, 8.2% B; 25–30 min, 80.3% A, 19.7% B; 30–35 min, 91.8% A, 8.2% B. Detection was performed at 280 nm and 320 nm. A standard curve was constructed using different gradient standard solutions of HPLC-grade punicalagin (Yuanye Bio-Technology Co., Ltd, Shanghai, China).

### 2.3 Antioxidant activity determinations

The DPPH radical scavenging was assessed following Williams et al.’s method (Williams et al., 1995). A 100 μL sample extract was added to a 2 mL of methanol solution of DPPH (6.25×10^−5^ M). After incubating the mixture in darkness for 20 min, the absorbance was measured at 517 nm using a spectrophotometer (UV-1800PC, MAPADA Instrument Co., LTD., Shanghai, China). The results were reported as μM Trolox equivalent antioxidant capacity (TEAC).

The FRAP reduction ability was determined based on Benzie et al.’s approach (Benzie and Strain, 1999). The TPTZ solution was prepared by combining 300 mM acetate buffer, 10 mM TPTZ solution (40 mM hydrochloric acid solution), and 20 mM ferric chloride solution at a volume ratio of 10:1:1. A 100 μL sample extract was sequentially mixed with 1 mL of distilled water and 1.8 mL of TPTZ solution. The reaction was conducted in a 37°C water bath for 10 min, and the absorbance was measured at 593 nm. The result was expressed as μM Trolox equivalent antioxidant capacity (TEAC).

### 2.4 Statistical analysis

Each experiment was performed in triplicates, and the results are reported as the mean ± standard deviation (SD). Data variance was analyzed using IBM SPSS Statistics 26 (SPSS Inc., Chicago, IL, USA) and the significance of differences was determined using the LSD method. The significance level was set at *p* < 0.05. GraphPad Prism 8 (GraphPad Software Llc., San Diego, CA, USA) and chiplot online (https://www.chiplot.online) were utilized for data visualization.

## 3 Results

### 3.1 Analysis of phenolic accumulation and antioxidant activity in pomegranate leaves

The color of pomegranate leaves transitions from tender red to tender green and then to deep green (**Supplementary Figure 1**). During leaf growth, TPC, TFC, TMAC, and punicalagin content in both pomegranate varieties exhibited a decreasing trend (**Table 1**). However, TFAC showed an increasing trend compared to other phenols. The TPC, TFC, and TFAC in leaves were 33.28–41.57 mg GAE/g FW (GAE, Gallic acid equivalent; FW, Fresh weight), 47.65–84.23 mg RE/g FW (RE, Rutin Equivalents), and 1.83–4.07 mg CE/g FW (CE, Catechin Equivalents), respectively. TMAC correlated with leaf color, when leaves were red at T1, TMAC was highest, with ‘Qingpi’ (694.71 nmol/g FW) significantly surpassing ‘Tunisia’ (647.98 nmol/g FW). As leaves matured, TMAC significantly decreased, reaching its lowest at T3 (4.91–6.05 nmol/g FW). Punicalagin content also decreased during leaf development, with the highest levels (10.88–11.25 mg/g FW) in young red leaves and the lowest levels (3.45–3.62 mg/g FW) in mature functional leaves. Antioxidant capacity of leaves, as determined by the DPPH method, was (395.25–545.25 μmol/g Trolox FW), and as determined by the FRAP method, it was (754.68–1349.58 μmol/g Trolox FW). Antioxidant capacity, TPC, TFC, and TMAC exhibited a consistent downward trend. Young leaves showed the highest antioxidant activity, while mature leaves displayed the lowest.

**Table 1:** Analysis of phenolic accumulation and antioxidant activity in pomegranate leaves throughout growth and development.

Parameter	Cultivar	T1	T2	T3
Total polyphenols (mg GAE/g FW)	Tunisia	40.74 ± 0.73 ab	38.45 ± 0.62 c	33.28 ± 0.39 d
	Qingpi	41.57 ± 0.42 a	38.86 ± 1.16 bc	33.78 ± 0.58 d
Total flavonoids (mg RE/g FW)	Tunisia	84.23 ± 0.63 a	79.44 ± 0.99 b	50.71 ± 0.95 d
	Qingpi	80.46 ± 0.55 b	65.56 ± 0.41 c	47.65 ± 0.70 e
Total flavanols (mg CE/g FW)	Tunisia	2.05 ± 0.08 c	2.15 ± 0.07 c	3.68 ± 0.12 b
	Qingpi	1.99 ± 0.09 c	1.83 ± 0.02 c	4.07 ± 0.25 a
Total anthocyanins (nmol/g FW)	Tunisia	647.98 ± 8.79 b	32.90 ± 0.50 c	4.91 ± 0.67 d
	Qingpi	694.71 ± 8.25 a	28.92 ± 0.43 c	6.05 ± 1.54 d
Punicalagin (mg/g FW)	Tunisia	10.88 ± 0.34 a	8.33 ± 0.06 b	3.62 ± 0.41 c
	Qingpi	11.25 ± 0.24 a	7.74 ± 0.26 b	3.45 ± 0.30 c
DPPH (μmol/g Trolox FW)	Tunisia	545.25 ± 6.12 a	481.28 ± 14.57 b	395.25 ± 4.31 c
	Qingpi	530.15 ± 9.12 a	482.13 ± 23.40 b	397.95 ± 3.11 c
FRAP (μmol/g Trolox FW)	Tunisia	1349.58 ± 2.40 a	1082.22 ± 8.53 b	758.61 ± 21.62 c
	Qingpi	1324.81 ± 12.42 a	1046.57 ± 9.02 b	754.68 ± 11.90 c

The values are expressed as the means ± SD. The leaves are categorized as T1 (red young leaves), T2 (tender green leaves), and T3 (deep green leaves). Different lowercase letters (a–c) indicate significant differences between sampling dates for each treatment, determined using Duncan’s multiple range test (p< 0.05).

### 3.2 Analysis of phenolic accumulation and antioxidant activity in pomegranate flowers

**Figure 1:**
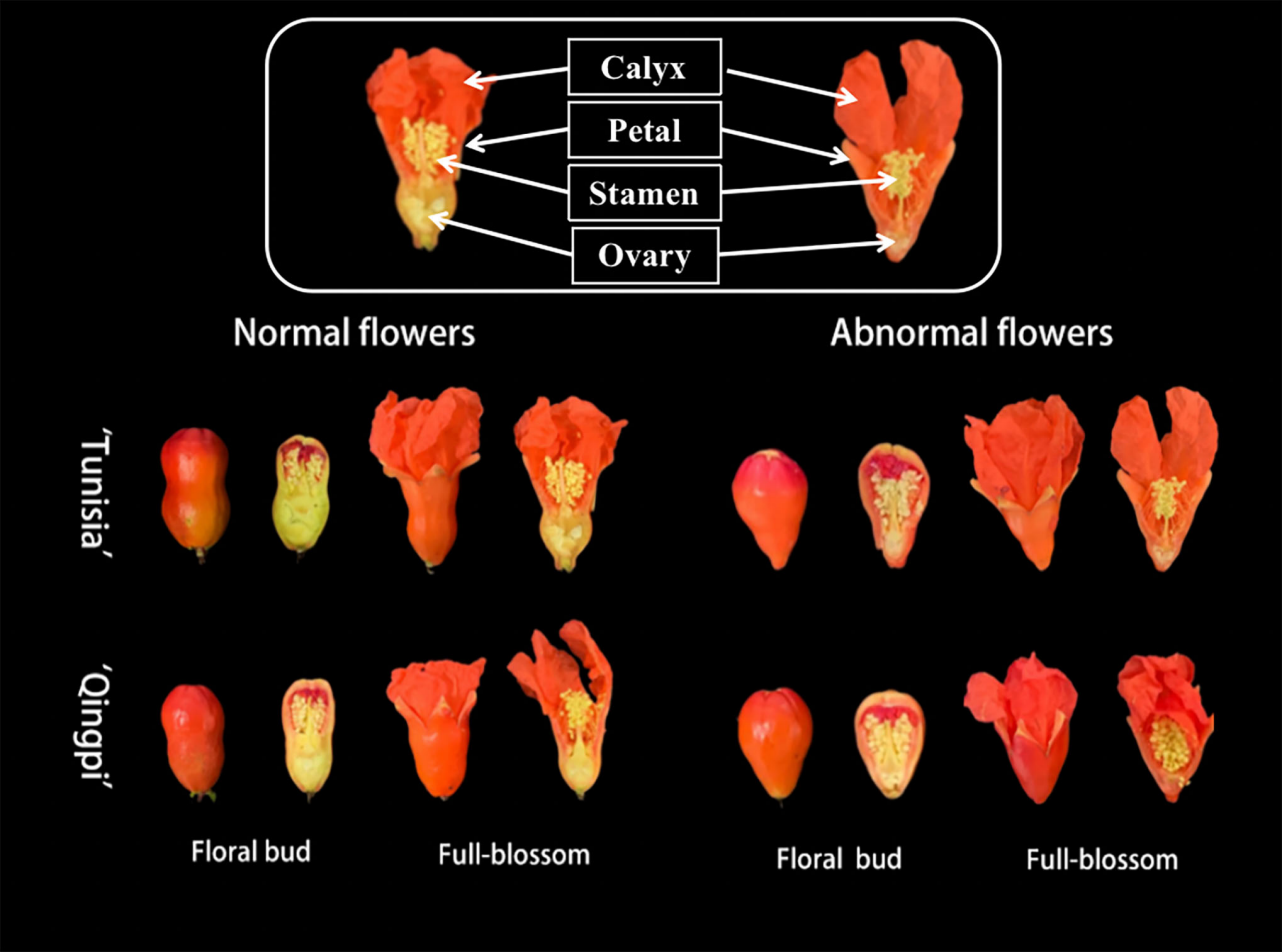
Different developmental stages of pomegranate flowers, highlighting the flower with the longitudinal profile. The flowers contain calyx, petal, stamen, and ovary.

The pomegranate flowers exhibit normal vase-shaped and abnormal bell-shaped structures (**Figure 1**). The phenolic substance content and antioxidant activity were analyzed in different parts of the pomegranate floral bud and fully blossomed flowers (**Table 2**). The TPC ranges in the calyx, petals, stamens, and ovary were 15.07–26.86 mg GAE/g FW, 18.86–50.15 mg GAE/g FW, 19.05–31.99 mg GAE/g FW, and 16.03–25.24 mg GAE/g FW, respectively. There were no significant differences in TPC between normal and abnormal flowers. The TPC decreased in the calyx, petal, and ovary during flower development, with significantly higher levels in the floral bud period compared to the full-blossom period. In contrast, the stamens showed an opposite trend, with lower TPC during the floral bud period than in the full-blossom period. The TPC in the petals was the highest, significantly higher than those in the calyx, stamen, and ovary.

**Table 2:** Phenolic accumulation analysis in pomegranate flowers throughout their growth and development.

Parameter	Part	Cultivar	Normal flower		Abnormal flower	
			Floral bud period	Full-blossom period	Floral bud period	Full-blossom period
Total polyphenols (mg GAE/g FW)	Calyx	Tunisia	26.33 ± 2.46 a	15.79 ± 0.91 b	26.86 ± 1.25 a	15.07 ± 0.96 b
		Qingpi	22.86 ± 0.81 a	16.86 ± 0.83 b	23.03 ± 2.40 a	16.86 ± 1.49 b
	Petal	Tunisia	49.40 ± 1.09 a	25.78 ± 1.43 c	50.15 ± 1.13 a	24.82 ± 1.41 c
		Qingpi	44.46 ± 0.29 b	18.86 ± 1.80 d	45.95 ± 0.85 ab	18.74 ± 2.75 d
	Stamen	Tunisia	22.83 ± 1.88 bc	31.99 ± 1.46 a	22.66 ± 1.72 bc	31.17 ± 0.57 a
		Qingpi	19.53 ± 0.37 c	26.39 ± 0.73 b	19.05 ± 1.49 c	26.05 ± 0.75 b
	Ovary	Tunisia	24.86 ± 0.90 a	17.28 ± 1.38 b	24.15 ± 0.70 a	17.90 ± 3.87 b
		Qingpi	25.24 ± 2.69 a	16.03 ± 1.22 b	25.08 ± 0.45 a	16.65 ± 1.95 b
Total flavonoids (mg RE/g FW)	Calyx	Tunisia	67.13 ± 1.24 b	44.63 ± 0.66 d	84.34 ± 1.16 a	45.16 ± 1.20 d
		Qingpi	55.44 ± 1.12 c	36.94 ± 0.92 e	66.45 ± 1.33 b	35.14 ± 0.80 e
	Petal	Tunisia	117.60 ± 1.24 a	56.93 ± 2.96 d	102.92 ± 2.51 b	49.05 ± 1.48 e
		Qingpi	98.69 ± 1.89 b	50.95 ± 3.46 de	82.11 ± 2.42 c	45.86 ± 1.35 e
	Stamen	Tunisia	74.19 ± 1.40 e	105.97 ± 0.78 a	83.33 ± 1.00 cd	88.11 ± 1.14 c
		Qingpi	59.39 ± 1.84 f	99.10 ± 2.58 b	75.02 ± 2.64 e	81.77 ± 1.91 d
	Ovary	Tunisia	87.89 ± 1.87 a	67.61 ± 1.96 c	84.57 ± 3.07 a	51.31 ± 1.56 e
		Qingpi	74.14 ± 0.97 b	60.16 ± 0.57 d	68.75 ± 2.28 bc	46.77 ± 0.54 e
Total flavanols (mg CE/g FW)	Calyx	Tunisia	0.37 ± 0.02 c	0.32 ± 0.01 c	0.58 ± 0.05 a	0.32 ± 0.01 c
		Qingpi	0.56 ± 0.01 ab	0.52 ± 0.02 b	0.58 ± 0.01a	0.35 ± 0.02 c
	Petal	Tunisia	0.71 ± 0.02 b	0.33 ± 0.02 c	0.70 ± 0.03 b	0.42 ± 0.02 c
		Qingpi	0.88 ± 0.06 a	0.35 ± 0.03 c	0.77 ± 0.03 b	0.39 ± 0.02 c
	Stamen	Tunisia	0.92 ± 0.03 e	1.10 ± 0.04 d	1.06 ± 0.01 de	1.26 ± 0.04 c
		Qingpi	1.04 ± 0.02 de	2.07 ± 0.04 a	1.70 ± 0.08 b	1.78 ± 0.04 b
	Ovary	Tunisia	0.56 ± 0.02 d	0.41 ± 0.01 d	0.93 ± 0.02 c	0.85 ± 0.05 c
		Qingpi	1.60 ± 0.23 b	1.38 ± 0.02 b	2.24 ± 0.09 a	2.08 ± 0.07 a
Total anthocyanins (nmol/g FW)	Calyx	Tunisia	163.94 ± 1.28 d	183.27 ± 3.58 c	114.95 ± 2.54 e	180.65 ± 2.19 c
		Qingpi	176.13 ± 5.83 c	227.91 ± 3.91 a	177.79 ± 2.16 c	217.65 ± 2.03 b
	Petal	Tunisia	1938.54 ± 1.60 a	1899.99 ± 6.15 b	1951.30 ± 3.29 a	1873.10 ± 11.05 b
		Qingpi	1929.43 ± 2.84 a	1792.47 ± 14.49 c	1938.39 ± 2.53 a	1780.47 ± 20.37 c
	Stamen	Tunisia	161.57 ± 6.19 f	272.95 ± 5.40 d	196.68 ± 8.28 e	459.51 ± 10.65 a
		Qingpi	151.37 ± 4.19 f	298.67 ± 5.20 c	158.66 ± 1.75 f	380.09 ± 4.96 b
	Ovary	Tunisia	49.47 ± 1.03 f	85.37 ± 1.20 d	167.91 ± 2.21 b	168.16 ± 0.90 b
		Qingpi	55.03 ± 1.97 e	129.82 ± 0.99 c	209.23 ± 3.48 a	211.31 ± 3.57 a
Punicalagin (mg/g FW)	Calyx	Tunisia	7.20 ± 0.07 b	6.48 ± 0.28 bc	3.98 ± 0.10 d	3.60 ± 0.02 d
		Qingpi	8.49 ± 0.04 a	5.83 ± 0.34 c	8.79 ± 0.16 a	6.84 ± 0.74 b
	Petal	Tunisia	45.58 ± 2.88 b	16.68 ± 0.13 e	55.50 ± 2.44 a	22.83 ± 0.17 d
		Qingpi	31.71 ± 1.41 c	13.78 ± 0.26 e	30.29 ± 2.19 c	16.28 ± 0.05 e
	Stamen	Tunisia	5.48 ± 0.10 c	6.21 ± 0.12 a	5.07 ± 0.07 d	5.54 ± 0.02 c
		Qingpi	4.92 ± 0.14 d	5.50 ± 0.14 c	5.40 ± 0.08 c	5.88 ± 0.11 b
	Ovary	Tunisia	13.13 ± 0.38 cd	10.12 ± 0.09 f	12.35 ± 0.03 de	8.66 ± 0.53 g
		Qingpi	16.53 ± 0.19 a	13.89 ± 0.34 c	14.88 ± 0.16 b	11.76 ± 0.26 e

Values (mean ± SD) of three replicates. Lowercase letters (a-c) in the table indicate significant differences between sampling dates for each treatment (p< 0.05) using Duncan’s multiple range test.

The TFC ranges in the calyx, petal, stamen, and ovary were 35.14–84.34 mg RE/g FW, 45.86–117.60 mg RE/g FW, 59.39–105.97 mg RE/g FW, and 46.77–87.89 mg RE/g FW, respectively. During flower development, the TFC significantly decreased in the calyx, petal, and ovary, while increasing in the stamens. The order of TFC during the floral bud period was petal > ovary > stamen > calyx, while during the full-blossom period, it was stamens > ovary > petals > calyx. Among normal flowers, the petals exhibited the highest TFC, while in abnormal flowers, the calyx and stamens showed the highest levels. The ‘Tunisia’ variety had significantly higher TFC than ‘Qingpi’.

**Table 2** demonstrates that the TFAC in the calyx, petal, stamen, and ovary were 0.37–0.58 mg CE/g FW, 0.33–0.88 mg CE/g FW, 0.92–1.70 mg CE/g FW, and 0.56–2.24 mg CE/g FW, respectively. Similar to TPC and TFC changes, TFAC in the calyx, petal, and ovary was higher during the floral bud period, while TFAC in the stamen increased during the full-blossom period. The TFAC in stamens of the ‘Tunisia’ variety was the highest (0.92–1.26 mg CE/g FW), whereas the calyx had the lowest levels (0.32–0.58 mg CE/g FW). The TFAC in the ovary of the ‘Qingpi’ variety was the highest (1.38–2.24 mg CE/g FW), while the calyx content was the lowest (0.34–0.58 mg CE/g FW). The ‘Qingpi’ variety flowers had higher TFAC than ‘Tunisia’.

The TMAC ranges in the calyx, petal, stamen, and ovary were 114.95–227.91 nmol/g FW, 1780.47–1951.30 nmol/g FW, 151.37–459.51 nmol/g FW, and 49.47–211.31 nmol/g FW, respectively. Stamens, calyxes, and ovaries had higher TMAC in full-blossom flowers, whereas it was higher for petals during the floral bud period. Moreover, the TMAC of abnormal flowers exceeded that of normal flowers.

The punicalagin contents in the calyx, petal, stamen, and ovary were 3.60–8.79 mg/g, 13.78– 55.50 mg/g, 4.92–6.21 mg/g, and 8.66–16.53 mg/g, respectively. Among different developmental stages, petals exhibited the highest punicalagin content, followed by ovaries, while calyxes and stamens had the lowest content. Notably, the punicalagin content in the petals of abnormal flowers from the ‘Tunisia’ pomegranate was significantly higher (55.50 mg/g) than that of normal flowers (45.58 mg/g). However, there was no significant difference in the punicalagin content between abnormal and normal flowers of the ‘Qingpi’ pomegranate, although it was significantly lower than that of the ‘Tunisia’ variety.

The antioxidant activity of ‘Tunisia’ and ‘Qingpi’ flower organs was determined using the DPPH and FRAP methods (**Table 3**). Both methods yielded consistent results. The antioxidant activity of petals, calyxes, and ovaries decreased as the flowers developed, whereas the antioxidant activity of stamens increased. No significant difference in antioxidant activity was observed between normal and abnormal flowers. During the floral bud period, petals and ovaries exhibited the highest antioxidant activity, while stamens demonstrated the highest antioxidant capacity during the full-blossom period.

**Table 3:** Antioxidant activity analysis in pomegranate flowers during growth and development.

Parameter	Part	Cultivar	Normal flower		Abnormal flower	
			Floral bud period	Full-blossom period	Floral bud period	Full-blossom period
DPPH (μmol/g Trolox FW)	Calyx	Tunisia	449.89 ± 6.03 ab	355.60 ± 4.51 c	458.59 ± 1.37 a	354.91 ± 7.44 cd
		Qingpi	431.09 ± 8.45 b	337.60 ± 4.87 cd	437.60 ± 4.48 b	334.12 ± 7.93 d
	Petal	Tunisia	509.27 ± 5.84 a	331.96 ± 4.61 b	522.99 ± 11.79 a	328.61 ± 8.61 b
		Qingpi	518.43 ± 2.13 a	342.37 ± 3.97 b	526.15 ± 11.65 a	340.19 ± 3.36 b
	Stamen	Tunisia	432.02 ± 2.79 c	489.16 ± 7.68 ab	428.77 ± 5.65 c	472.90 ± 2.46 b
		Qingpi	424.92 ± 5.67 c	494.60 ± 1.35 a	415.05 ± 7.45 c	477.09 ± 9.15 ab
	Ovary	Tunisia	522.61 ± 0.81 a	419.76 ± 4.08 e	516.10 ± 2.82 a	411.58 ± 4.43 e
		Qingpi	492.88 ± 6.25 b	476.15 ± 1.23 c	488.93 ± 4.49 bc	442.71 ± 6.70 d
FRAP (μmol/g Trolox FW)	Calyx	Tunisia	930.55 ± 6.83 a	576.89 ± 1.70 c	934.35 ± 5.50 a	575.22 ± 11.87 c
		Qingpi	832.63 ± 3.69 b	544.55 ± 15.34 d	842.50 ± 9.51 b	541.15 ± 3.43 d
	Petal	Tunisia	1500.76 ± 3.04 a	677.52 ± 3.32 d	1505.78 ± 10.63 a	670.22 ± 3.66 d
		Qingpi	1310.70 ± 22.40 c	620.78 ± 11.04 e	1375.41 ± 1.85 b	609.66 ± 7.23 e
	Stamen	Tunisia	804.53 ± 2.65 c	981.64 ± 7.89 a	802.72 ± 16.39 c	970.04 ± 16.85 a
		Qingpi	730.78 ± 4.79 d	963.18 ± 18.25 ab	717.03 ± 6.49 d	930.78 ± 5.30 b
	Ovary	Tunisia	962.81 ± 30.71 a	647.37 ± 9.31 c	964.25 ± 5.15 a	628.55 ± 9.34 c
		Qingpi	846.52 ± 6.75 b	570.22 ± 14.83 d	838.74 ± 7.11 b	571.56 ± 4.83 d

Values of three replicates are expressed as the means ± SD. Different lowercase letters (a-c) in the table indicate significant differences between sampling dates for each treatment (p< 0.05) using Duncan’s multiple range test.

### 3.3 Analysis of phenolic accumulation and antioxidant activity during pomegranate fruit development

**Figure 2:**
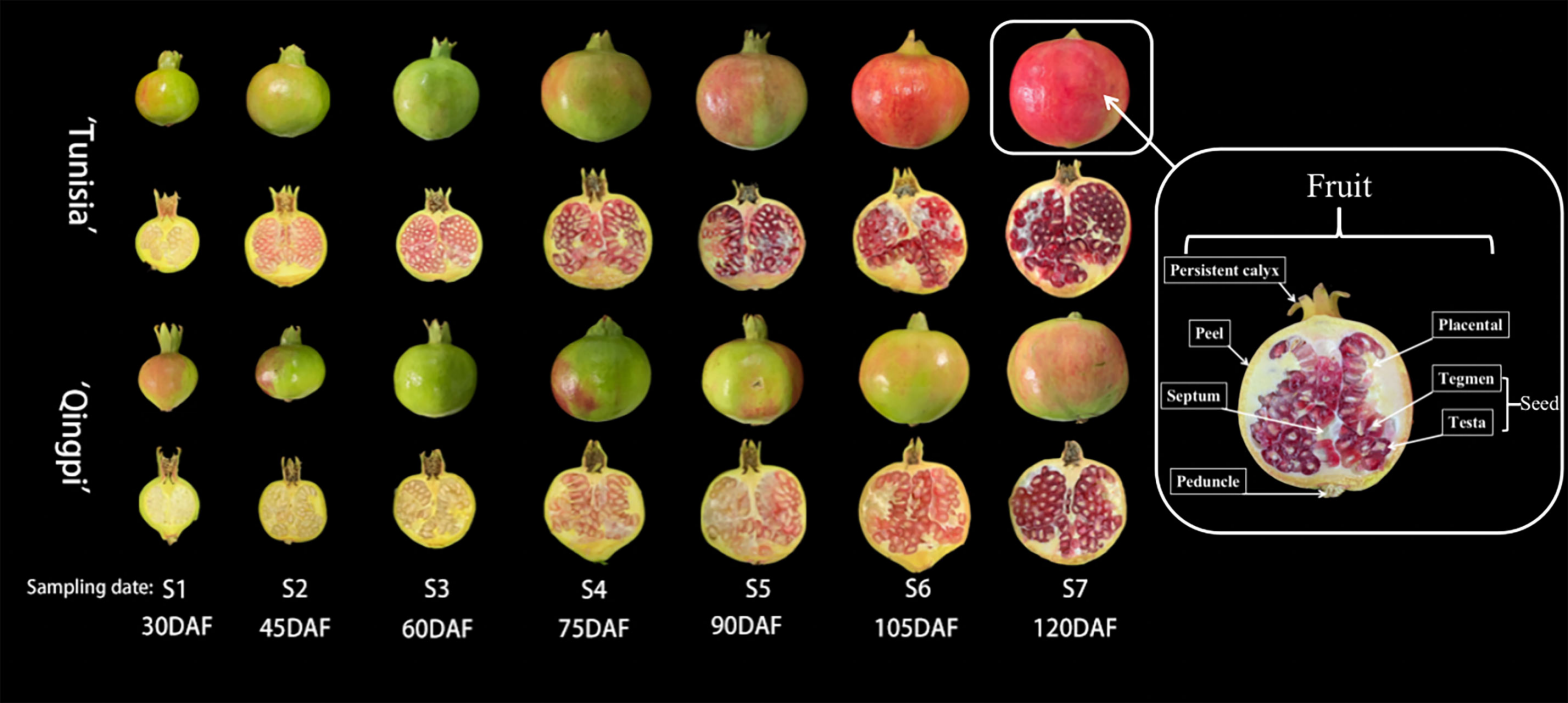
Pomegranate fruit undergoes various developmental stages, comprising the inner and outer parts. It includes the persistent calyx, peduncle, peel, placenta, septum, and seed. The seed consists of tegmen and testa, with the fleshy part serving as the juice source.

**Figure 3:**
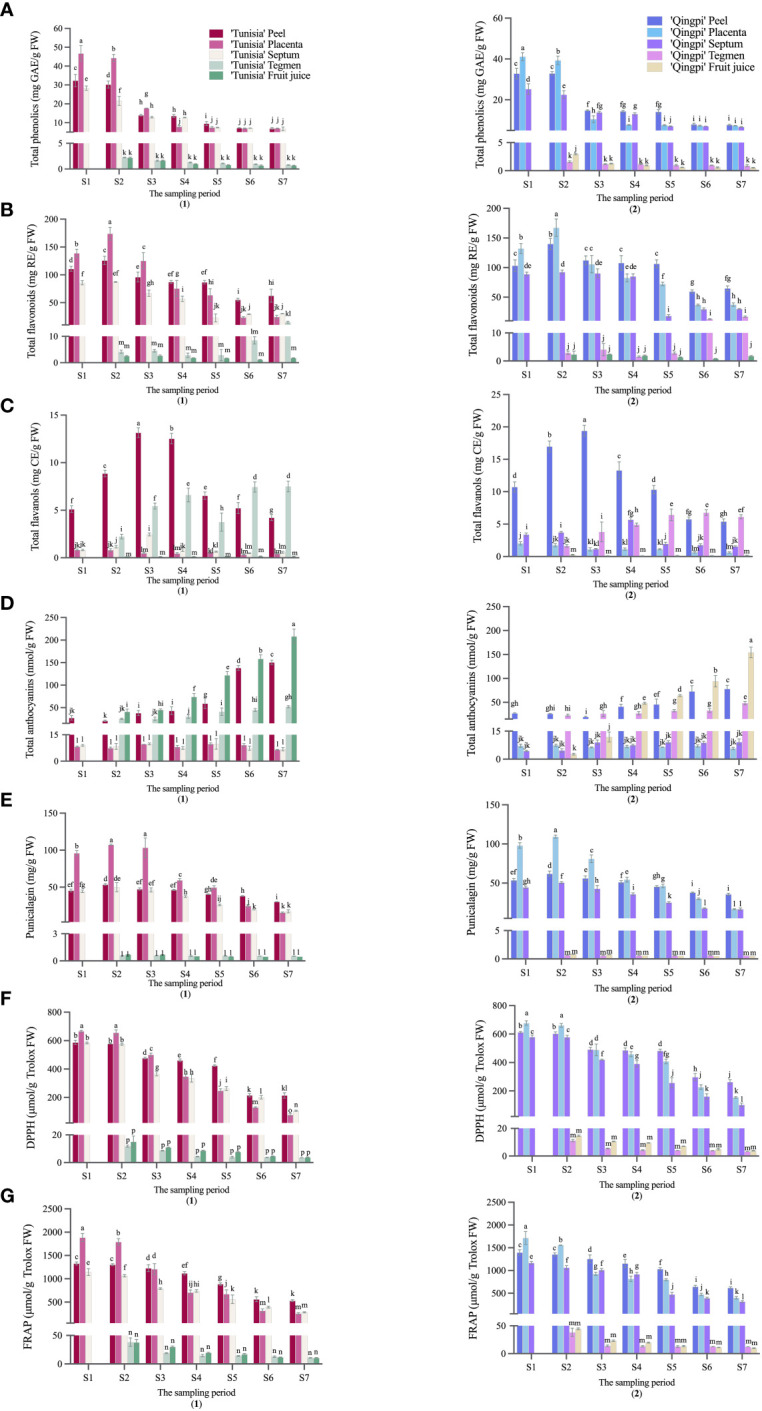
Phenolic accumulation and antioxidant activity analysis in pomegranate fruit during growth and development was conducted. The parameters measured were TPC **(A)**, TFC **(B)**, TFAC **(C)**, TMAC **(D)**, Punicalagin **(E)**, DPPH **(F)**, and FRAP **(G)** for ‘Tunisia’ (1) and ‘Qingpi’ (2). Mean ± standard deviation (n = 3) was presented in each column, and a *t*-test was performed at the 0.05 significance level (*p*< 0.05). Different letters (a-p) indicate significant differences, while the same letter indicates no significant difference.

The developmental stages of pomegranate fruit are shown in **Figure 2**. The pomegranate fruit peel color changes from red to green and then back to red. ‘Tunisia’ fruit pulp begins coloring at S2, while ‘Qingpi’ starts at S4. The TPC decreases during fruit development (**Figures 3A1, A2**). The TPC of peel, placenta, and septum decreases rapidly from S2 to S3. The TPC of tegmens and juice decrease gradually, and are significantly lower than the other parts. Initially, the TPC order was placenta > peel > septum > tegmen ≈ juice. In the middle and late stages, there’s no significant difference among peel, placenta, and septum, but they’re significantly higher than juice and tegmen. ‘Tunisia’ has higher TPC than ‘Qingpi’.

During pomegranate development, the TFC initially increases, then decreases, and finally increases slowly (**Figures 3B1, B2**). In the early stage, the TFC in peel, placenta, and septum were higher. At S2 stage, the TFC was highest in peel, placenta, and septum (55.42–140.02 mg RE/g FW, 23.75–173.98 mg RE/g FW, 18.33–92.61 mg RE/g FW, respectively). The TFC in tegmens was highest during fruit ripening (‘Tunisia’, 14.97 mg RE/g FW; ‘Qingpi’, 17.09 mg RE/g FW). Placenta has the highest TFC in the early stage, while peel has the highest in the late stage. Juice and tegmens have significantly lower TFC than other parts at the same stage.

**Figures 3C1, C2** show the distribution of TFAC in different parts of the pomegranate fruit during development. The TFAC was mainly found in peels and tegmens. Peel TFAC increases, peaks in the S3 period, then decreases. Tegmens exhibit significant changes, with the highest TFAC at fruit maturity.

The TMAC is mainly distributed in peel, tegmen, and juice. The TMAC increases during development and peaks at fruit maturity (**Figures 3D1, D2**). ‘Tunisia’ has the fastest TMAC accumulation from S4 to S5, while ‘Qingpi’ accumulates fastest from S6 to S7. TMAC during the mature period was 3–57 times higher than the early stage. The order of TMAC concentration was juice > peel > tegmen > placenta ≈ septum. ‘Tunisia’ has a higher TMAC than ‘Qingpi’.

Punicalagin is primarily found in the placenta, peel, and septum of the pomegranate fruit, with lower contents in their juice and tegmen. The punicalagin content in peel, placenta, and septum initially increases, reached the peak at the S2-stage, then declines, and was the lowest in the mature period (S7). In the premiddle-stage, the order of punicalagin concentration was placenta > peel > septum > tegmen ≈ juice. In the mature stage, it was peel > placenta > septum > tegmen > juice.

The antioxidant activity of pomegranate during development was evaluated using DPPH (**Figure 3F**) and FRAP methods (**Figure 3G**). Overall, the fruit exhibited a decreasing trend in antioxidant activity. Notably, the peel, placenta, and septum demonstrated higher antioxidant activities, while the tegmens and juice showed significantly lower levels. Initially, the placenta exhibited the highest antioxidant activity, followed by the peel and septum. However, starting from stage S4, the placenta’s antioxidant activity rapidly declined, and the peel had the highest activity, followed by the placenta and septum. Throughout all stages, the tegmens and juice consistently displayed the lowest antioxidant activity.

### 3.4 Comparison of antioxidant activity and phenolic content in different parts of pomegranate during development

**Figure 4:**
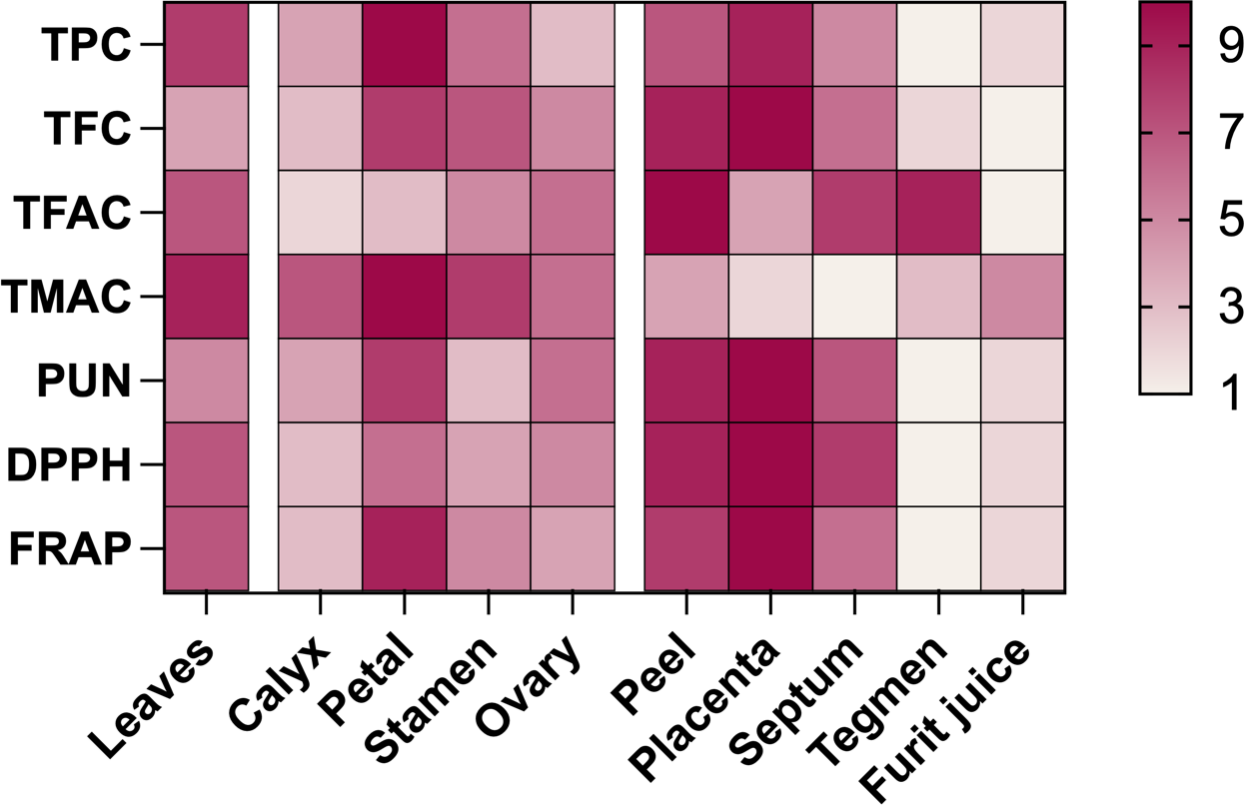
Comparing antioxidant activity and phenolic content in various parts of developing pomegranates. Color depth represents concentration, with red indicating high and white indicating low. Values range from 1 to 10. TPC, Total polyphenols; TFC, total flavonoids; TFAC, total flavanols; TMAC, total anthocyanins; PUN, punicalagin.

During pomegranate development, phenolic substances and antioxidant activity were compared across different parts (**Figure 4**), and values were assigned on a scale of 1 to 10 based on concentration. Results revealed that the placenta had the highest contents of TFC and punicalagin, the peel had the highest TFAC, and the petals had the highest TPC and TMAC. The order of antioxidant activity was placenta > peel >petals ≈ leaves. The fruit’s edible part was primarily protected by the high phenolic content and strong antioxidant activity of the placenta, peel, and septum. Anthocyanin, along with total flavanols, plays a significant role in pomegranate juice as the main pigment and antioxidant.

### 3.5 Correlation analysis between antioxidant activity and phenolic content in different organs of pomegranate during development

**Figure 5:**
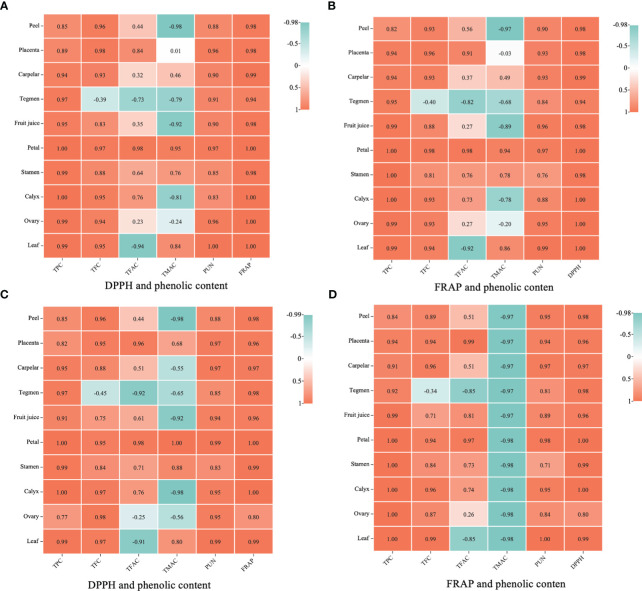
Correlation analysis between antioxidant activity and phenolic content in different tissues and organs of *Punica granatum*. The concentration was expressed by the color depth, orange means high, blue means low, and the value from low to high was 1–10. **(A, B)**: ‘Tunisia’; **(C, D)**: ‘Qingpi’; TPC, Total polyphenols; TFC, total flavonoids; TFAC, total flavanols; TMAC, total anthocyanins; PUN, punicalagin.

The antioxidant activity showed a significant positive correlation with phenolic content in various organs of pomegranate (**Figure 5**). The correlation coefficient between DPPH and FRAP (**Figures 5A, C**) and FRAP (**Figures 5B, D**) was 0.94–1.00. Furthermore, the antioxidant activity of pomegranate organs was positively correlated with TPC, punicalagin, and TFC. Except for the fruit’s tegmen, the antioxidant activity in different organs showed a significant positive correlation with TFC, with correlation coefficients being 0.71–0.99. The correlation coefficients between antioxidant activity and TPC and punicalagin were 0.77–1.00 and 0.71–1.00, respectively. The relationship between antioxidant activity and TFAC and TMAC varied across different parts. The TFAC showed significant positive correlations with the antioxidant activity of placenta, tegmen, petal, stamen, calyx, and leaf, and the correlation coefficient were respectively 0.84 ~ 0.99 (Positive correlation, P), -0.92 ~ -0.73 (Negative correlation, N), 0.97 ~ 0.98 (P), 0.64 ~ 0.76 (P), 0.73 ~ 0.76 (P) and -0.94 ~ -0.85(N). The TMAC showed significant correlations with the antioxidant activity of peel, juice, petal, stamen, calyx, and leaf, while other parts of the pomegranate did not show significance. The antioxidant activity of the petal showed a particularly high correlation coefficient of 1 with TMAC.

## 4 Discussion

Pomegranate was widely utilized in food production and processing. Its edible part, the testa, can be consumed fresh or used to make fruit juice. Pomegranate juice was a significant source of anthocyanins, which possess health benefits due to their antioxidant properties (Gardeli et al., 2019). Besides the fruit juice, other parts of the pomegranate, such as the peel, leaves, and flowers, are abundant in phenols (Tzulker et al., 2007; Legua et al., 2012; Bekir et al., 2013a). These parts exhibit high antioxidant activity and hold great value for development and utilization.

Pomegranate leaves are rich in bioactive compounds and serve as a natural source of pigments and antioxidants. Yu et al. (2021) characterized the phenolic composition and antioxidant activity of several medicinal and food plants, finding that pomegranate leaves have the highest TFC (199.26 mg GAE/g DW, DW, Dry Weight) and antioxidant activity compared to other varieties. The leaves can be used as a substitute for tea, extract development, and synthetic antioxidants (Wang et al., 2013; Mansour et al., 2022). Pomegranate leaf tea contains more phenolic substances (41.01-83.43 mg/100 g) than green tea (Legua et al., 2012), which is lower than the TPC (33.28-41.57 mg/g FW) measured in the leaves of this study. Our study shows that the phenolic substances and antioxidant activity of pomegranate leaves are highest when they are red (T1), which is similar to the study by Zhang et al. (2010). Therefore, for tea or other beverages made from pomegranate leaves, the nutritional value was higher when young leaves are harvested compared to mature leaves.

Edible flowers have gained popularity in recent years due to their unique flavors and nutritional content. Flowers from peach (Li and Wang, 2011), broccoli (Lou et al., 2023), chrysanthemum (Wei et al., 2023), and *Osmanthus fragrans* (Wang et al., 2022) are used as a good source for food products. Pomegranate flowers, with their vibrant colors and high phenolic content, surpass white roses, chrysanthemums, and other edible flowers, making them desirable in the consumer market (Teniente et al., 2023). Bekir et al. (2013b) measured the highest TPC, TFC, TMAC in various pomegranate dry flowers as 330.9 mg GAE/g DW, 29.5 mg QE/g DW (QE, quercetin equivalent), and 0.70 mg CGE/g DW (CGE, cyanidin-3-glucoside equivalent), respectively. In our study, the highest TPC, TFC, and TMAC in pomegranate fresh flowers were 50.15 mg GAE/g FW, 117.6 mg RE/g FW, and 1938 nmol/g FW, respectively, significantly surpassing the levels in fruit juice. Interestingly, studies have shown that the proportion of abnormal flowers (bell-shaped flowers) in the total flowers can reach 60–94.88%, with abnormal flowers being good for pollination but not for fruit yield (Chen et al., 2017). This study found that abnormal flowers are rich in phenolic substances similar to normal flowers. Although abnormal flowers are typically discarded as bio-waste, their long harvesting period highlights their potential as a local specialty food and as raw materials for phenolic substance extraction and processing, thereby enhancing the economic benefits of pomegranate.

Pomegranate products, including juice, wine, vinegar, and others, are widely accepted by consumers globally (Kalaycıoğlu and Erim, 2017). Pomegranate juice, constituting 78% (*w/w*) (Singh et al., 2022) of fresh food products, exhibits three times higher antioxidant activity compared to wine and green tea (Arantino et al., 2023). In this study, the TPC, TFC and TMAC of ‘Tunisia’ and ‘Qingpi’ fruit juice were respectively 0.53-2.95 mg GAE/g, 0.84-2.55 mg RE/g, and 2.67-208.35 nmol/g, which were lower than ‘Wonderful’ pomegranate juice (Arantino et al., 2023). El Kar et al. (2011) reported TPC, TFC, and TMAC in juice of ‘Tunisia’ and ‘Gabsi’ as 458-3299 mg GAE/L, 135-636 mg QE/L, and 11-178 mg CGE/L, respectively, and their ranges were also higher than those in this experiment. Except for the edible part, the pomegranate peel comprising 40–50% of the total fruit weight was considered a rich source for the extraction of bioactive substances due to its abundant phenolic content (El Kar et al., 2011; Akhtar et al., 2015). Despite its high phenolic content, the utilization of pomegranate peel as a by-product remains limited, leading to wastage (Lampakis et al., 2021; Montefusco et al., 2021). Liu et al. (2022) discovered that punicalagin content was highest in the peel. Additionally, Derakhshan et al. (2018) showed that pomegranate peel exhibits higher antioxidant activity and phenolic content than fruit juice and tegmens, with a positive correlation between phenolic content and antioxidant activity, these are consistent with the results of this study. In addition, in our study, the fruit placenta exhibited the highest TPC, TFC, punicalagin contents, and antioxidant activity during the premiddle period of fruit development.

The TPC, TFC, TFAC, and antioxidant activity of pomegranate flowers and fruits peaked in the early growth stage. As different organs develop, the content of phenolic compounds and antioxidant activity gradually decrease, which was consistent with the study of Mirdehghan and Rahemi (2007). Conversely, the TFC of pomegranate fruit tegmens and pomegranate flower stamens increased with fruit development, suggesting variations in flavonoid accumulation among different organs. Punicalagin, a specific component of pomegranate, exhibits therapeutic effects on cervical cancer (Teniente et al., 2023). Punicalagin content was the highest in pomegranate peel, and the granatine content decreased gradually with the growth and development of pomegranate (Bekir et al., 2013a). This study detected punicalagin in different parts of pomegranate the placenta contained the highest punicalagin (109.30 mg/g FW).

The antioxidant activity of various pomegranate organs correlates with changes in phenolic substances. Initially, during early fruit development, different organs exhibited higher phenol content and stronger antioxidant activity. However, as the pomegranate matured, the DPPH and FRAP levels in pomegranate leaf, flower, and fruit decreased progressively as the fruit ripened, consistent with findings in *Rosa rugosa* (Zeng and Peng, 2007) and apple leaves and fruit (Wojdyło and Oszmiański, 2020). Montefusco et al. (2021) demonstrated a positive correlation between TFC and antioxidant activity during pomegranate growth. Similarly, Gil et al. (2000) determined that punicalagin contributed to 50% of the antioxidant activity in pomegranate juice, supporting the present study’s results. The antioxidant activity of both pomegranate varieties positively correlated with TPC, TFC, and punicalagin, indicating that these compounds primarily contribute to pomegranate’s high antioxidant capacity. Derakhshan et al. (2018) reported that phenolic content and antioxidant activity in pomegranate fruit follow the order: peel > seed > juice. However, the analysis of various pomegranate fruit parts revealed a different order of the concentration of phenolic content and antioxidant activity: placenta > peel, septum > tegmen > juice.

## 5 Conclusion

This study revealed that pomegranate leaves, flowers, and fruits exhibit high TPC and are abundant in TFC, TMAC, and punicalagin. These components offer various health benefits, making pomegranate a valuable source of phenols. Notably, the pomegranate placenta highlighted the highest TPC, TFC, punicalagin content, and antioxidant activity compared to other fruit parts like juice and tegmens. As a widely cultivated fruit tree globally, pomegranate serves as an excellent raw material for food processing. To enhance utilization rates and reduce costs, priority should be given to developing and utilizing phenols from floral bud flowers, young leaves, and young thinning fruits. Mature fruit peel, juice, and full-bloom flowers are suitable for anthocyanin development and utilization. The placenta and peel of young fruits can be utilized for punicalagin extraction. This study contributes new insights into processing and utilizing pomegranate leaves, flowers, and fruits, particularly their non-edible parts.

## Data availability statement

The original contributions presented in the study are included in the article/**Supplementary Material**. Further inquiries can be directed to the corresponding author.

## Author contributions

HZ: Investigation, Conceptualization, Funding acquisition, Writing – review & editing. MW: Data curation, Formal Analysis, Methodology, Software, Writing – original draft. GY: Conceptualization, Investigation, Methodology, Writing – original draft. JP: Writing – review & editing. KT: Writing – review & editing. XT: Writing – review & editing. YD: Writing – review & editing. HW: Writing – original draft, Resources. JH: Writing – original draft, Resources. XL: Writing – review & editing, Methodology. LL: Writing – review & editing, Methodology. QD: Funding acquisition, Writing – review & editing, Conceptualization.
