# Supplementary material for: Comparative analysis of the phenolic contents and antioxidant activities of different parts of two pomegranate (Punica granatum L.) Cultivars: ‘Tunisia’ and ‘Qingpi’
Source: Front Plant Sci. 2023 Sep 29;14:1265018. doi: 10.3389/fpls.2023.1265018 (PMC10570729; doi:10.3389/fpls.2023.1265018)
Supplement: Supplementary file 1 [file DataSheet_1.docx]

Supplementary Material

Comparative Analysis of the Phenolic Contents and Antioxidant Activities of Different Parts of Two Pomegranate (*Punica granatum* L.) Cultivars

Huifen Zhang^1+^, Miao Wang^1+^, Guoqiang Yu^1^, Jing Pu^1^, Kun Tian^1^, Xiaofu Tang^1^, Ying Du^1^, Hongxia Wu^2^, Jiong Hu^3^, Xian Luo^1^，Lijin Lin^1^ and Qunxian Deng^1*^

^1^College of Horticulture, Sichuan Agricultural University, ChengDu, Sichuan, China

^2^Rural Professional Technology Association of Huili, Huili, Sichuan, China

^3^Science and Technology Association of Huili, Huili, Sichuan, China

^+^ These authors contributed equally to this work

**^*^ Correspondence:**

 Qunxian Deng

[dqxlwj@sina.com.cn](mailto:dqxlwj@sina.com.cn)

# Supplementary Figure


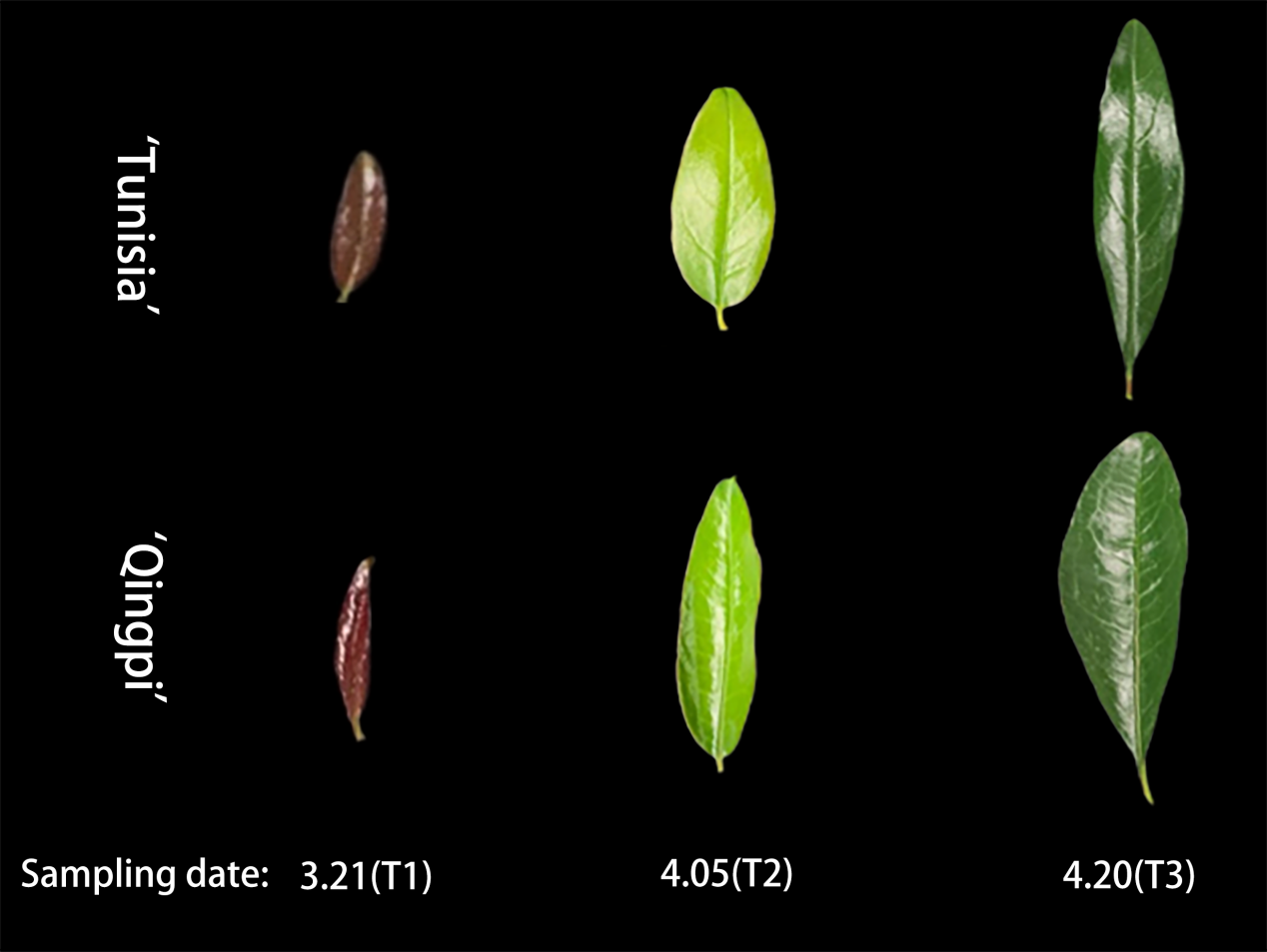


Supplementary Figure 1 Different developmental stages of pomegranate leaves. The leaves are categorized as T1 (red young leaves), T2 (tender green leaves), and T3 (deep green leaves).
